# Data silos are undermining drug development and failing rare disease patients

**DOI:** 10.1186/s13023-021-01806-4

**Published:** 2021-04-07

**Authors:** Nathan Denton, Monique Molloy, Samantha Charleston, Craig Lipset, Jonathan Hirsch, Andrew E. Mulberg, Paul Howard, Eric D. Marsh

**Affiliations:** 1 Gene Therapy Program, Department of Medicine, Perelman School of Medicine, University of Pennsylvania, Philadelphia, PA 19104 USA; 2 Department of Medicine, Orphan Disease Center, Perelman School of Medicine, University of Pennsylvania, Philadelphia, PA 19104 USA; 3 Clinical Innovation Partners, New York, NY USA; 4 Syapse, San Francisco, CA USA; 5 Bios Ventures, San Francisco, CA USA; 6 Neurogene Inc., New York, NY USA; 7 Amicus Therapeutics, Philadelphia, PA 19104 USA; 8 Departments of Neurology and Pediatrics, Children’s Hospital of Philadelphia, Perelman School of Medicine, University of Pennsylvania, Philadelphia, PA 19104 USA; 9 Division of Neurology, Children’s Hospital of Philadelphia, Philadelphia, PA 19104 USA

**Keywords:** Rare diseases, Drug development, Knowledge bases, Registries, Research design, Data management

## Abstract

Data silos are proliferating while research and development activity explode following genetic and immunological advances for many clinically described disorders with previously unknown etiologies. The latter event has inspired optimism in the patient, clinical, and research communities that disease-specific treatments are on the way. However, we fear the tendency of various stakeholders to balkanize databases in proprietary formats, driven by current economic and academic incentives, will inevitably fragment the expanding knowledge base and undermine current and future research efforts to develop much-needed treatments. The proliferation of proprietary databases, compounded by a paucity of meaningful outcome measures and/or good natural history data, slows our ability to generate scalable solutions to benefit chronically underserved patient populations in ways that would translate to more common diseases. The current research and development landscape sets too many projects up for unnecessary failure, particularly in the rare disease sphere, and does a grave disservice to highly vulnerable patients. This system also encourages the collection of redundant data in uncoordinated parallel studies and registries to ultimately delay or deny potential treatments for ostensibly tractable diseases; it also promotes the waste of precious time, energy, and resources. Groups at the National Institutes of Health and Food and Drug Administration have started programs to address these issues. However, we and many others feel there should be significantly more discussion of how to coordinate and scale registry efforts. Such discourse aims to reduce needless complexity and duplication of efforts, as well as promote a pre-competitive knowledge ecosystem for rare disease drug development that cultivates and accelerates innovation.

## Context

I didn’t know when all of this started that we would enroll in a bunch of natural history studies, and trials, and genetic studies, that were all not going to be coordinated. I thought the data would be shared. I thought it would be used to understand patient journeys, how does a child go from being healthy to progressing in this disease state, …how we were going to use that data [to help] the next generation of ‘Savannah’s’. We learned from trial and error that these trials were uncoordinated, and that Savannah was just another specimen in these uncoordinated studies… The system that was supposed to be helping us was actually hurting us. We were learning very little about her disease…it was not a learning system, it was a box-checking system.

Dr. Tracy Dixon-Salazar, PhD, neuroscientist, geneticist, and parent to Savannah, a patient living with Lennox-Gastaut Syndrome.

## Commentary

Despite its name and highly specific manifestations, rare diseases collectively affect a huge number of people—approximately 3.5–5.9% of the world’s population (~ 263 to 466 million), according to a recent estimate [1]. Moreover, this substantial global community is acutely affected by issues that are systemic to general biomedical research. The rare disease community is currently experiencing a substantial increase in clinical and research activities due to considerable progress in our understanding of the fundamental biology and etiology of many clinically described disorders, mainly due to advances in genetic medicine. This new understanding of specific mechanisms, combined with emerging tools like genetically based therapies, forms the basis for “precision” or targeted therapeutic approaches for many rare genetic diseases and has inspired optimism in the patient, clinical, and research communities. The notable successes of several gene therapy programs, such as for spinal muscular atrophy and retinal dystrophy, have helped to advance growing research efforts to characterize the basic biology and to enable novel therapeutics development. Coincident with this basic biological research, clinicians, patients, and researchers are working to quantify the clinical manifestations of the many rare diseases through registries, natural history studies, and other patient-focused data collections.

Despite these scientific advances, however, considerable challenges with rare disease drug development remain. This difficulty can be largely attributed to small, heterogeneous, and geographically dispersed patient populations that limit the ability to develop precise outcome measures and document natural history data. This paucity of good quality outcome and natural history data impedes our ability to apply targeted treatments to precise genotype/phenotype combinations and meaningfully assess their clinical effects. Small, complex patient populations are also often ill-suited to many key design and power principles required for traditional clinical development programs, such as statistically persuasive Phase 3 clinical trials. Far from being unique to this community, our advancing understanding indicates that such hallmarks typically associated with rare diseases are highly relevant to many common diseases (e.g., cancer and cardiovascular disease). This is due to the increasing identification of disease subcategories that share common symptoms but may also possess discrete pathological mechanisms that require precision-guided treatments.

We now find ourselves at a crossroads at which scientific and therapeutic advances for many rare genetic disorders are possible, but for which knowledge of disease-specific clinical manifestations and logistical considerations are limiting factors. We have not arrived at this unfavorable situation through a lack of ingenuity, technology, or even funding. Instead, it has emerged from academic, pharmaceutical, and patient groups operating within the long-standing system of economic and academic incentives in which proprietary databases, patient cohorts, and novel approaches are leveraged to protect funding streams and nascent intellectual property. While this is a reality for all disorders, the rare disease community is struck particularly hard by numerous complex problems because many life-threatening rare diseases affect children (thus raising issues for consent, especially for enrolment in multiple studies) and their study can involve invasive data collection procedures (e.g., bone marrow biopsy). There is therefore an ethical imperative to devise data collection standards and sharing practices which minimize redundant testing while maximizing the utility of collected data to support this highly vulnerable population.

Lack of access to patient data lessens the value of any specific program because individual companies and institutions are unable to accumulate a critical mass of knowledge that can substantially de-risk drug development. A lack of meaningful, specific top-down guidance can also render large-scale data collecting efforts futile in the context of meeting regulatory standards for the development of novel products. In short, all stakeholders can contribute various pieces of a biological puzzle that can illuminate the full spectrum of disease when made available in shareable states.

The NIH [2] and FDA [3] are critical stakeholders in the development of rare disease therapies. The NIH supports critical basic research into the biology and etiology of rare diseases through the National Center for Advancing Translational Sciences (NCATS [4]), which “develops, demonstrates, and disseminates innovations that reduce, remove, or bypass system-wide bottlenecks in the translational process” in specific programs such as the Rare Disease Registry Program, RaDaR [5]. In 2020, as part of the reorganization of the Office of New Drugs (OND), the FDA also created a new rare disease hub in the Center for Drug Evaluation and Research (CDER), called the Division of Rare Diseases and Medical Genetics. This new Division seeks to “coordinate research, collaboration, and communication for rare diseases policy and programming” across the agency. FDA funding also supports the Rare Disease Cures Accelerator-Data and Analytics Platform (RDCA-DAP [6]) and the generation of guidance documents on the rare disease drug development process. We commend the FDA and the NIH for continuing to address the unique needs of the rare disease community. However, unfortunate organizational silos exist that make it difficult to standardize approaches for modernizing rare disease drug development.

It follows that some of the biggest threats to progress can be attributed to data silos, exclusive access rights, and an inconsistent or reluctant adoption of sharing practices. Despite some successes, the current biomedical research and development ecosystem is far from fulfilling its therapeutic, academic, or economic potential. Fragmentation of fundamental disease knowledge, including natural history studies and registries, also imposes growing costs on patients and caregivers who find it increasingly burdensome and difficult to participate in ostensibly redundant clinical research. Most rare diseases are serious, life-limiting or life-threatening disorders that substantially impact patients’ and their families’ lives, for which effective therapeutics are needed in a timely manner. We have an obligation to use and share patient data responsibly; coordination amongst all the researchers, foundations, and families for rare diseases can ensure more efficient and effective collection of this data to ultimately reduce the burden on families and accelerate the development of more effective treatments by promoting the best use of our most precious resources—patients’ time, limited energy, and goodwill.

There is an urgent imperative to rectify this situation. In addition to rare disease patients’ and their family’s race against time to obtain effective therapies, we are also in the midst of massive data proliferation. Technology and open data rules (including meaningful use regulations requiring interoperability and patients’ ability to share their electronic medical records) are democratizing registries such that many patient communities have initiated registries and deployed data collection tools, including wearable sensors, apps, and social media channels. Rare disease “umbrella” groups (that is, those that work across many rare diseases) have initiated larger registry and data initiatives (for example, see programs by NORD, Rare-X, and RDCA-DAP) which further expand the registry ecosystem. While we applaud these efforts and their intent, the rapid expansion of registries is poised to exacerbate data loss, replication, and/or data gaps without standard practices changing among stakeholders.

We also have a generational opportunity to harness, support, and attempt to validate the use of emerging technologies like artificial intelligence and machine learning to enable data analysis on an unprecedented scale [7]. Realizing this potential is contingent on clinical meaningfulness being established though, which takes time, energy, and careful thought to analyze hordes of data. Developing high-quality, transparent data sets can allow researchers to seize this unique, potentially revolutionary opportunity to leverage multiple data sources and make the most of each patient’s experience. The rare disease space represents the ideal environment to apply the lessons from projects like the COVID-19 Evidence Accelerator [8] to hone the deployment of data-gathering infrastructure and analytics through clear mechanisms for data sharing and collaboration. Other potential solutions could involve the NIH leading a meeting addressing the incentives across academia and industry, and/or Congress prioritizing and requiring publicly funded research be standardized and shared as part of the U.S. national artificial intelligence strategy to make data sharing and data use easier and more accessible [9]. Such efforts would provide direct, immediate benefit to chronically underserved patient populations; they would also provide a roadmap of principles and practices that can be applied to almost any other disease with significant unmet medical need to cultivate and accelerate innovation that improves human health.

Well-designed patient registries and natural history studies are key tools in the study and development of more effective new treatments for rare diseases because they allow us to reduce unexplained variation and generate more specific, testable hypotheses. For example, the tuberous sclerosis complex (TSC) foundation, in conjunction with academics and funded by various industry partners, has generated a long-term natural history registry that enabled the creation of a disease-modifying trial. If successful, the outcome of this trial could result in substantial improvement in cognition and behaviors in children with TSC [10]; the natural history registry contributed data and insight that allowed this trial to happen. Similar approaches to data sharing by a number of registries for other disorders such as juvenile rheumatoid arthritis (CARRA [11]), cystic fibrosis (CF Foundation [12]) and RDCA DAP (Critical Path Institute [6]) have enabled similar successes in advancing our understanding of disease pathology and helping to address unmet medical needs. While some guidance on rare disease research has been issued by the FDA recently, however, the utility and effectiveness of such instruments in the broader research ecosystem are hampered by a general lack of top-down guidance from regulators and the hoarding mentality encouraged by the current set of incentives.

There are clear benefits to all stakeholders collaborating. Sharing in the development of standardized clinical data elements will enhance our collective baseline understanding of the clinical features of any rare disease and lay the foundation for innovation and competition on novel analytics, outcome measures, and quantifiable tools for both research and improvements in clinical care. Ironically, the lack of generalizable knowledge makes it more likely that individual data holders overestimate the value of the data they have because they do not know what data they are missing. Its siloed nature often means that data collected at great difficulty and expense by many well-meaning actors proves inadequate for building successful drug development efforts and guiding regulatory decision-making. Until we create better regulatory and business incentives for both top-down data coordination and bottom-up data generation, we will wander in a sea of data, but find that very little of it satisfies our thirst for innovation—in both the rare and common disease spaces.

## Conclusion

The advent of potentially curative genomic technologies, along with advances in computing power and analytics, provides an opportune time to start a broad dialogue and reach consensus between stakeholders on key issues relating to registry design, data sharing, and data governance. Pulling together the currently fragmented rare disease research and development ecosystem has the potential to provide a fuller understanding of disease manifestations from genotype to phenotype to benefit a substantial underserved patient population. Such principles and practices have the potential to transform biomedical research and improve human health by cultivating both top-down standard setting and bottom-up collaboration in gathering data and accelerating innovation of new analytic, diagnostic, and therapeutic tools.

## Data Availability

Not applicable.

## Abbreviations

CARRA: Childhood Arthritis and Rheumatology Research Alliance
CDER: Center for Drug Evaluation and Research
CF: Cystic fibrosis
FDA: Food and Drug Administration
NCATS: National Center for Advancing Translational Sciences
NIH: National Institutes of Health
NORD: National Organization for Rare Disorders
ODC: University of Pennsylvania’s Orphan Disease Center
OND: Office of New Drugs
RaDaR: Rare Disease Registry Program
RDCA-DAP: Rare Disease Cures Accelerator-Data and Analytics Platform
TSC: Tuberous sclerosis complex
